# Imbalanced spectral data analysis using data augmentation based on the generative adversarial network

**DOI:** 10.1038/s41598-024-63285-4

**Published:** 2024-06-09

**Authors:** Jihoon Chung, Junru Zhang, Amirul Islam Saimon, Yang Liu, Blake N. Johnson, Zhenyu Kong

**Affiliations:** 1 Department of Industrial Engineering, Pusan National University, Busan, South Korea; 2 Grado Department of Industrial and Systems Engineering, Virginia Tech, Blacksburg, VA USA

**Keywords:** Bioinformatics, High-throughput screening

## Abstract

Spectroscopic techniques generate one-dimensional spectra with distinct peaks and specific widths in the frequency domain. These features act as unique identities for material characteristics. Deep neural networks (DNNs) has recently been considered a powerful tool for automatically categorizing experimental spectra data by supervised classification to evaluate material characteristics. However, most existing work assumes balanced spectral data among various classes in the training data, contrary to actual experiments, where the spectral data is usually imbalanced. The imbalanced training data deteriorates the supervised classification performance, hindering understanding of the phase behavior, specifically, sol-gel transition (gelation) of soft materials and glycomaterials. To address this issue, this paper applies a novel data augmentation method based on a generative adversarial network (GAN) proposed by the authors in their prior work. To demonstrate the effectiveness of the proposed method, the actual imbalanced spectral data from Pluronic F-127 hydrogel and Alpha-Cyclodextrin hydrogel are used to classify the phases of data. Specifically, our approach improves 8.8%, 6.4%, and 6.2% of the performance of the existing data augmentation methods regarding the classifier’s F-score, Precision, and Recall on average, respectively. Specifically, our method consists of three DNNs: the generator, discriminator, and classifier. The method generates samples that are not only authentic but emphasize the differentiation between material characteristics to provide balanced training data, improving the classification results. Based on these validated results, we expect the method’s broader applications in addressing imbalanced measurement data across diverse domains in materials science and chemical engineering.

## Introduction

Spectroscopic technologies such as X-ray diffraction (XRD), Nuclear Magnetic Resonance (NMR), Raman scattering, and Electrical Impedance Spectral (EIS) are fundamental tools for the characterization of experimental samples in chemistry and materials science. XRD has found extensive use throughout industry and research laboratories for more than a century^1^. It is proven to be an effective method for characterizing crystalline materials as it captures detailed information on the long-range periodic nature of crystal structures. In contrast, NMR and Raman measurements are more strongly dependent on localized chemical interactions and are widely used to characterize the structure of molecular materials^2,3^. EIS is a technique used to determine the impedance characteristics of an electrochemical interface. It has been used increasingly in biomaterials studies to understand the interactions between the surface and the biological environment. While their mechanisms and uses may vary, all of these spectroscopic methods generate comparable one-dimensional spectra consisting of unique peak positions, widths, and intensities. These features often serve as “fingerprints” for material characteristics, including patterns and phases^4,5^. Identification of the characteristics of unknown specimens can be achieved by comparing newly measured spectra with those of established materials in experimental databases^6,7^. However, the analysis process is complicated by factors such as measurement noise, background signals, and inherent minor deviations in the spectra^8^. To automate this process, machine learning has recently emerged as an effective tool since it can automatically classify experimental spectra along material characteristics with significant accuracies^9,10^.

The popular method within the domain of machine learning is deep neural networks (DNNs). These networks consist of several layers of artificial neurons designed to mimic the structure and functioning of the human brain^11^. DNNs is widely used in classification tasks of spectral data as they can automatically extract discriminating features. Specifically, DNNs is utilized for supervised classification methods since these methods can use the label information of each class (i.e., material characteristics of spectral data), providing accurate classification results. For example, Kantz et al.^12^ used DNNs to classify Liquid Chromatography-Mass Spectrometry (LC-MS) spectral peak shapes. This approach improves peak filtering performance by reducing the false peaks by more than 90% compared to the traditional chemometric methods. Zeng et al.^13^ utilized one-dimensional convolutional neural network (CNN) to classify the visible-near infrared spectra of corn seed to evaluate seed viability. In addition, Lee et al.^14^ developed a CNN-based model to classify interested phases from a mixture of inorganic compounds using XRD. Similarly, Schuetzke et al.^8^ built a robust CNN model for automatically classifying phases using the XRD patterns. This shows superior performance in automatic phase identification of cement compounds and iron ores. These studies assumed balanced training spectral data between classes (i.e., material characteristics of spectral data) in their supervised classification methods.

However, the balanced spectral data among the classes is difficult to appear in actual chemistry, physics, and industries generating the spectral data. For example, medical diagnostic applications often generate imbalanced spectral data reflecting the common asymmetry encountered in health status among screened individuals (e.g., more true negatives than true positives are typically encountered in preventative diagnostics). Materials science and chemistry applications also often generate imbalanced spectral data reflecting the common asymmetry of composition–process–structure–property relations, such as associated with phase equilibrium (e.g., the physics governing the thermodynamics of mixtures often results in asymmetric distributions of stable, unstable, and transition states with respect to varying mixture composition). For example, it is common to encounter samples of one type in accelerated materials discovery applications based on the unknown structure of a material design space and the initially selected search parameters, which may be done randomly or based on prior knowledge. As such, imbalanced spectral data is inevitably generated mainly in actual experiments and industries. However, the imbalanced spectral data leads to compromised supervised classification performance using DNNs. Specifically, the prediction in classification models tends to be biased towards the majority class, which has sizable spectral data samples. This leads to a high probability of misclassifying samples from the minority class^15^.

To address this significant challenge arising from imbalanced spectral data in classification utilizing DNNs, a viable solution is to employ data augmentation techniques to create a balanced training dataset across spectral data of different material characteristics. Basic data augmentation methods, including rotation, flipping, synthetic minority oversampling technique (SMOTE)^16^, and Borderline-SMOTE (B-SMOTE)^17^ are commonly used for balancing training data within the classification due to their straightforward implementation^18–20^. However, these techniques primarily take into account localized information, thus failing to capture the complete data distribution and address the challenge of overfitting^21,22^. Consequently, these methods are unsuitable for generating realistic spectral data with various characteristics^23,24^. In contrast, there has been a growing trend in the active utilization of Generative Adversarial Networks (GAN) and its variations^25,26^, including deep convolutional GAN (DCGAN)^27^, CDRAGAN^28^, and Covid GAN^29^, to supplement the limited actual data because of the GAN’s capacity to generate authentic data by comprehensively learning the entire data distribution of actual data through two neural networks: the discriminator and the generator^30,31^. Specifically, Balancing GAN (BAGAN)^32^ is a well-known GAN-based method focusing on generating minority class samples. Huang and Jafari^28^ proposed an enhanced version of BAGAN (BAGAN-GP)^28^ by providing an improved initialization method and gradient penalty technique to stabilize the training process. Based on the GAN’s capacity, it has been widely used in spectral data analysis. For example, Wu et al.^33^ used a GAN framework to augment synthetic Raman spectroscopy data of skin cancer tissue to address the difficulties of class imbalance in the context of cancer tissue data. Similarly, Gao et al.^34^ utilized GAN to generate seizure events in long-term EEG spectra to overcome the data imbalance problem for accurate classification.

Although these studies generate realistic spectral data to provide balanced data among the various material characteristics, they do not consider generating the samples enabling differentiation between characteristics (i.e., characteristics-distinguishable samples). The characteristics-distinguishable samples can further improve the classification performance, which is the ultimate goal of generating the data in the spectral data analysis. The samples can be generated by joint optimization between GAN and the classifier. Specifically, the classifier guides the generator in GAN to create samples that could improve classification results. Regarding this direction, we proposed a novel data augmentation method in a recent paper^15^ that jointly optimizes between GAN and the classifier with several stabilizing techniques. The method validated its effectiveness in imbalanced data in additive manufacturing processes. Therefore, we apply the method to spectral data to address the imbalanced spectral data issue that commonly occurs in actual experiments and industries. In this paper, the effectiveness of our method is validated by using the spectral data collected from actual experimentation. Specifically, the electrical impedance spectral data from Pluronic F-127 hydrogel and Alpha-Cyclodextrin hydrogel are used. The phases of spectral data are provided as imbalanced. The results show that the imbalanced spectral data can be successfully overcome by our method in the classification of the phases. In particular, our approach enhances the F-score, Precision, and Recall of the classifier by an average of 8.8%, 6.5%, and 6.2%, respectively, compared to the benchmark methods. Moreover, the technique has great generality. Thus, it can be further applied to address the classification with imbalanced spectral data in other material science or chemical engineering domains.

## Results

Several real-world case studies are provided to show the effectiveness of our method in imbalanced spectral data analysis. In “Case study using spectral data from Pluronic F-127 hydrogel” and “Case study using spectral data from Alpha-Cyclodextrin hydrogel” sections, comparative case studies involving benchmark methods are provided. Specifically, spectral data from two actual materials, Pluronic F-127 hydrogel, and Alpha-Cyclodextrin hydrogel, are provided in “Case study using spectral data from Pluronic F-127 hydrogel” and “Case study using spectral data from Alpha-Cyclodextrin hydrogel” sections, respectively. The imbalanced spectral data regarding the material phases are provided to evaluate the performance. Therefore, the material characteristics that need to be classified are the material phases in the case studies. The performance assessment is conducted based on the classification results obtained from the imbalanced training dataset. All case studies utilize the Keras with TensorFlow backend. The experiments are carried out on an NVIDIA Tesla P4 GPU within the Google Colab environment^35^.

### Benchmark methods

Regarding the benchmark methods, both sampling-based and GAN-based approaches are used. Within the sampling-based category, two techniques that SMOTE^16^ and B-SMOTE^17^ are used. These methods are implemented using the Python imbalanced-learn library. For the GAN-based approaches, three state-of-the-art class-conditional GAN methods, namely, CDRAGAN^28^, BAGAN-GP^28^, and Covid GAN^29^ are selected. In addition, Cooperative GAN^36^, which is also class-conditional GAN that jointly optimizes GAN and the classifier without stabilizing technique, is utilized as one of the benchmark methods. Beyond the GAN methods, we also considered the diffusion model^37^, which has been widely used recently because of its superior generative performance. Specifically, the class-conditioned U-Net-based diffusion model (CCD-diffusion)^38,39^ is used as a benchmark method. Finally, the baseline is established by evaluating the classification performance without employing any data augmentation method.

### Performance evaluation measure

The performance assessment is determined by the classifier’s F-score, Precision, and Recall^40^. Convolutional neural network (CNN) is used as a classifier. The F-score expressed in Eq. (1) is a composite metric that combines both Precision and Recall.

1 $$\begin{aligned} \text {F-score} = 2\times \frac{\text {Precision} \times \text {Recall}}{\text {Precision}+\text {Recall}}. \end{aligned}$$

As the primary goal of this paper is to enhance classification accuracy using imbalanced training data, it includes case studies that encompass different balanced ratios. A balanced ratio refers to the proportion between the training data size of the minority and majority classes. Each case study is iterated ten times. The performance measure is the average performance across all classes from the ten repetitions.

### Case study using spectral data from Pluronic F-127 hydrogel

**Figure 1:**
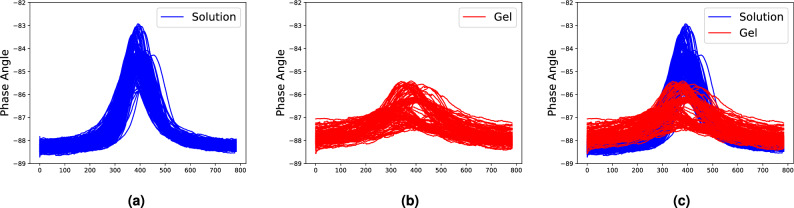
Spectral data of Pluronic F-127 hydrogel from (**a**) solution; (**b**) gel; (**c**) solution and gel.

Pluronic F-127 (PF-127), a nonionic amphiphilic surfactant, demonstrates a reversible thermogelling process in aqueous solutions, resembling the behavior observed in other Pluronic compounds^41^. In this section, PF-127 hydrogel libraries are used for the case study. It’s been widely used and studied in a wide range of applications. 96 PF-127 deionized water mixtures with different mass ratios are formulated in the 96-well plates. The concentration of PF-127 deionized water varies from 0.3125 to 30 wt% with an increment of 0.3125 wt%. The phase angle-frequency spectrum of each sample is collected by a sensor-based high-throughput method. The collected spectra are labeled as solution or gel to study the composition-property relationships of PF-127 hydrogels. Three repeated experiments provide 288 spectral data. Specifically, 181 spectral data of solution (Fig. 1a) and 107 of gel (Fig. 1b) are utilized for the case study. The frequency range for each experiment and concentration is determined by the spectrum width. Moreover, different sensors are employed in repeated experiments, resulting in diverse spectrum frequency ranges. To use all the spectrum data from three experiments, the x-axis of spectrum data is converted into the sequence of sensor measurements (from one to eight hundred, which is the length of data). The detailed data collection procedure and frequency range of each experiment are described in “Data collection of Pluronic F-127 hydrogel libraries” section.

Table 1 describes the imbalanced training data, where the balanced ratios between the two phases are 0.013, 0.027, and 0.039, respectively. The ratio is set because balanced ratios below 0.013 result in significantly poor performance for the classifier. The remaining data sets are used as testing data.

**Table 1:** Imbalanced training data samples in Pluronic F-127 hydrogel case studies.

Majority class	Minority class	Balanced ratio	Majority class training samples	Minority class training samples
Solution	Gel	0.013	150	2
Solution	Gel	0.027	150	4
Solution	Gel	0.039	150	6

**Figure 2:**
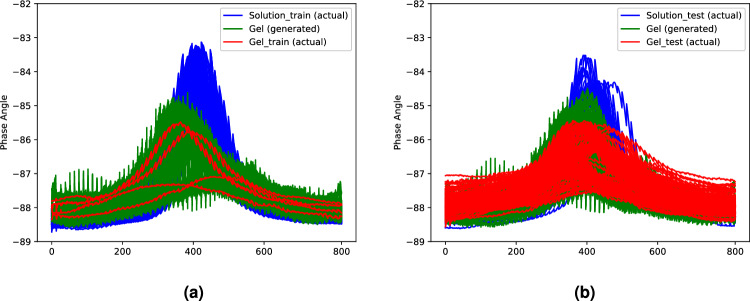
Comparison between generated data of Pluronic F-127 hydrogel with (**a**) actual training data; (**b**) actual testing data when the balanced ratio is 0.027.

Figure 2 shows the actual and generated samples from the proposed method, respectively. Specifically, Fig. 2a describes the actual imbalanced training data in Table 1, while Fig. 2b represents the actual testing data. The generated samples in Fig. 2 when the balanced ratio is 0.027 are realistic spectral data with apparent differences between phases achieved through a learning process in our method. Specifically, the results show that the generated samples from our approach successfully learn the features of the test data of the gel phase (Fig. 2b) from the small number of training data samples (Fig. 2a).

**Figure 3:**
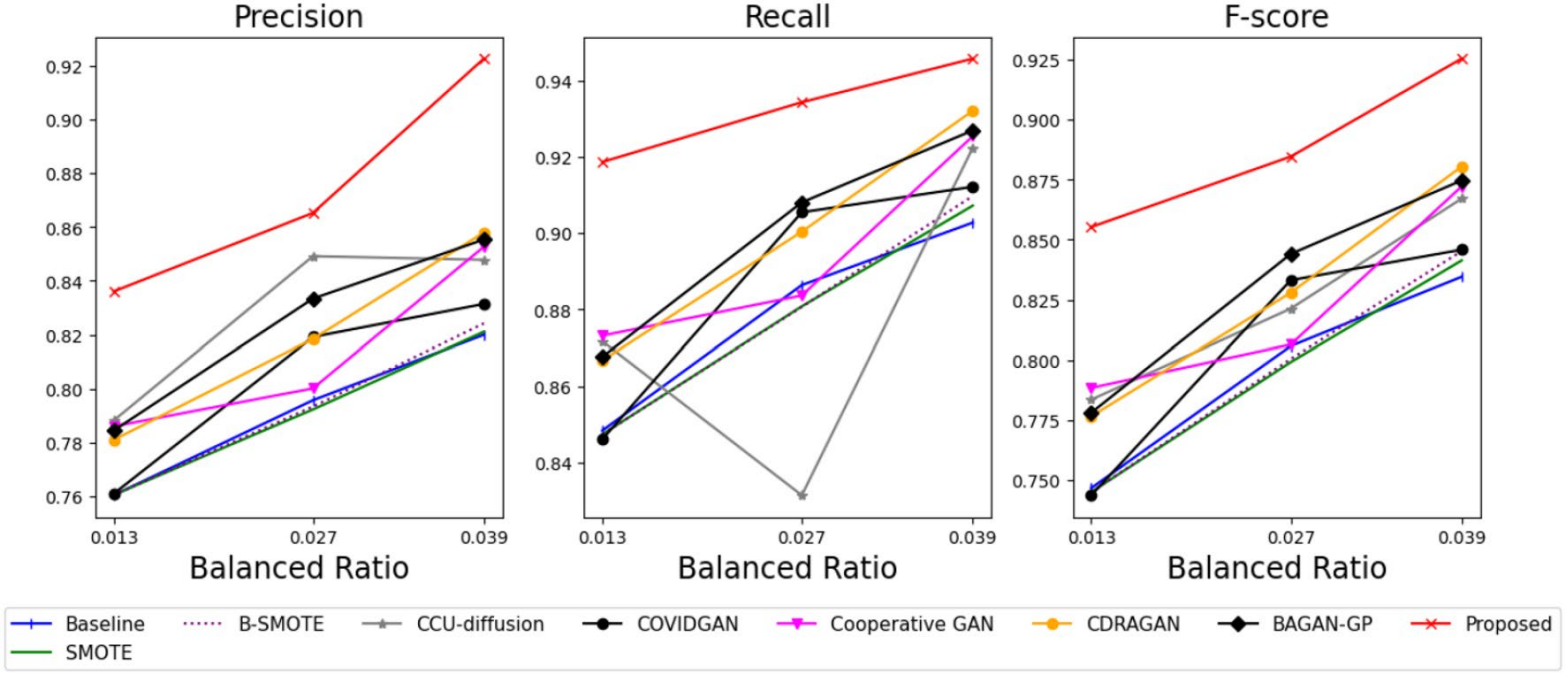
Performance evaluation using Pluronic F-127 hydrogel with several balanced ratios.

Figure 3 shows the performance evaluation of the benchmark and our methods using the generated samples from each method. The detailed averages and standard deviations of the performance of each method are provided in Appendix 1.3. Compared to a baseline result that uses only imbalanced data as training data of the classifier, the sampling-based methods, including B-SMOTE^17^ and SMOTE^16^, tend to exhibit similar or worse performance. This is because the small number of minority class samples prevents the generation of various data from sampling-based methods.

Conversely, GAN-based approaches typically outperform sampling-based methods because their generators learn the actual distribution of samples from minority classes and generate diverse training data for the classifier. In particular, the generator from our method provides more diverse and better-quality samples than other GAN-based methods by jointly optimizing the classifier with stabilizing techniques, resulting in improvements in classification results. Specifically, our method improves 9.4%, 8.3%, and 5.3% of the average performance of the benchmark methods regarding their F-score, Precision, and Recall, respectively. To check the significance of the proposed method over the benchmark methods, we performed the paired-T test^42^ between the proposed method and a benchmark method, achieving the best F-score performance, the composite metric of precision and recall. Specifically, Cooperative GAN, BAGAN-GP, and CDARAGAN show the best performance among the benchmark methods at the balanced ratios of 0.013, 0.027, and 0.039, respectively. Table 2 illustrates that the proposed method shows statistically significant improvements over the best benchmark method at a 95% significance level in most cases. Furthermore, Table 3 represents the average training time from each of the data augmentation methods. Although the proposed method takes a relatively large training time compared to benchmark methods, it is valuable to use the proposed method to achieve significant improvements in classification results over the benchmark methods.

**Table 2:** P-value of statistical hypothesis test in Pluronic F-127 hydrogel case studies.

Alternative hypothesis	Balanced ratio	Precision	Recall	F-score
Proposed $\ge$ Cooperative GAN	0.013	0.002	0.004	0.002
Proposed $\ge$ BAGAN-GP	0.027	0.049	0.049	0.046
Proposed $\ge$ CDRAGAN	0.039	0.004	0.120	0.022

**Table 3:** On average training time of each method in Pluronic F-127 hydrogel case studies.

Method	Time (min)	Method	Time (min)	Method	Time (min)	Method	Time (min)
Proposed	24	Cooperative GAN	18	COVID GAN	5	SMOTE	2
BAGAN-GP	23	CDRAGAN	21	CCU-diffusion	18	BSMOTE	2

**Figure 4:**
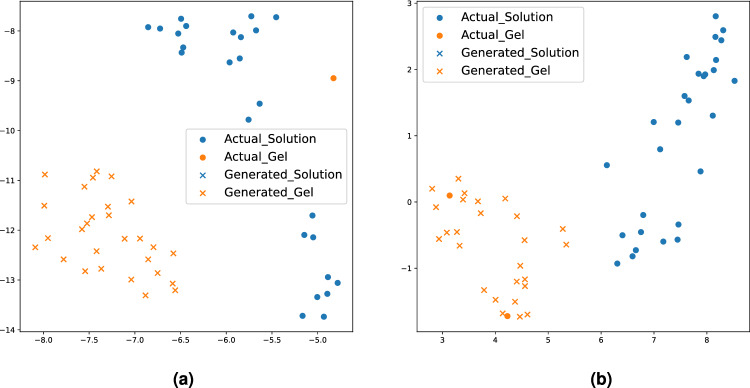
t-SNE of the feature from the intermediate layer of the classifier from our method in epochs (**a**) 0 and (**b**) 140 when the balanced ratio is 0.027.

Figure 4 illustrates the efficacy of the generated samples produced by our approach by comparing their features in the classifier with those of actual samples when the balanced ratio is 0.027. Specifically, Fig. 4 displays the t-distributed Stochastic Neighbourhood Embedding (t-SNE) of the feature extracted from the intermediate layer of our method’s classifier. t-SNE is a nonlinear dimensionality reduction technique designed for visualizing high-dimensional data by projecting it into lower-dimensional spaces^43^. In Fig. 4, ‘$\bullet$’ represents t-SNE of the features from the intermediate layer of classifiers extracted from actual samples, while ‘$\times$’ represents features from the generated samples within the balanced training batch. To achieve a balanced training batch, there is an abundance of ‘$\times$’ instances for the minority class (i.e., the gel phase) in each batch. In Fig. 4a, it is evident that the distribution patterns between actual and generated samples are distinct at epoch 0. Specifically, the ‘$\bullet$’ of the gel phase is not aligned with ‘$\times$’ of its phase. Furthermore, it is aligned with the ‘$\bullet$’ of the solution phase. Because our approach is designed to generate realistic and distinguishable samples between the phases, the features extracted from the generated samples (denoted as ‘$\times$’) accurately align with those from the actual samples (represented as ‘$\bullet$’) based on their respective phases at epoch 140 (Fig. 4b). Furthermore, the features associated with each phase are distinctly separated. This observation confirms the realistic and phase-discriminative characteristics of the generated samples produced by our method. By employing balanced training data characterized by these attributes, our method attains a high level of classification performance.

### Case study using spectral data from Alpha-Cyclodextrin hydrogel

**Figure 5:**
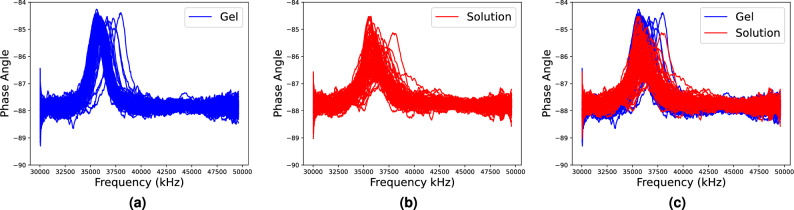
Spectral data of alpha-Cyclodextrin hydrogel from (**a**) gel; (**b**) solution; (**c**) gel and solution.

Alpha-Cyclodextrin based polypseudorotaxane supramolecular hydrogels, which are based on the self-assembly of a polymer chain “guest” and Alpha-Cyclodextrin “host”, are promising materials for a wide range of applications, including drug delivery and tissue engineering^44^. In this section, hydrogel libraries of Alpha-Cyclodextrin ($\alpha$-CD)/Polyethylene glycol (PEG) are used for the case study. It’s known that composition plays a vital role in forming hydrogels. Here, 96 $\alpha$-CD/PEG hydrogel samples with different mass ratios of $\alpha$-CD to PEG are formulated in the 96-well plate. The concentration of PEG is kept at 120 mg/mL while the concentration of $\alpha$-CD varies from 20 to 40 mg/mL. The phase angle-frequency spectrum of each sample is collected by a sensor-based high throughput method. The collected spectra are labeled as solution or gel to study the composition-structure relationship of $\alpha$-CD/PEG hydrogels. Three repeated experiments offer 288 spectral data. Specifically, 194 spectral data of gel (Fig. 5a) and 94 of solution (Fig. 5b) are provided for the case study. The detailed procedure of data collection is described in “Data collection of Alphasps Cyclodextrin hydrogel libraries” section.

Table 4 illustrates the training data with various balanced ratios. Specifically, the balanced ratios that the classifier’s performances are applicable in practice are utilized. The remaining samples in each phase are used as testing data.

**Table 4:** Imbalanced training data samples in Alpha-Cyclodextrin hydrogel case studies.

Majority class	Minority class	Balanced ratio	Majority Class training samples	Minority class training samples
Gel	Solution	0.025	120	3
Gel	Solution	0.050	120	6
Gel	Solution	0.083	120	10

**Figure 6:**
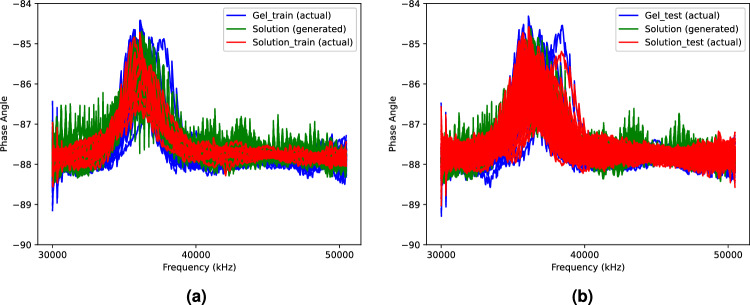
Comparison between generated data of Alpha-Cyclodextrin hydrogel with (**a**) actual training data; (**b**) actual testing data when the balanced ratio is 0.050.

Figure 6 shows the samples of actual and generated samples from the proposed method when the balanced ratio is 0.050. Similar to Fig. 2, the generated samples from our approach successfully learn the features of the test data of the solution phase (Fig. 6b) from the small number of training data samples (Fig. 6a).

**Figure 7:**
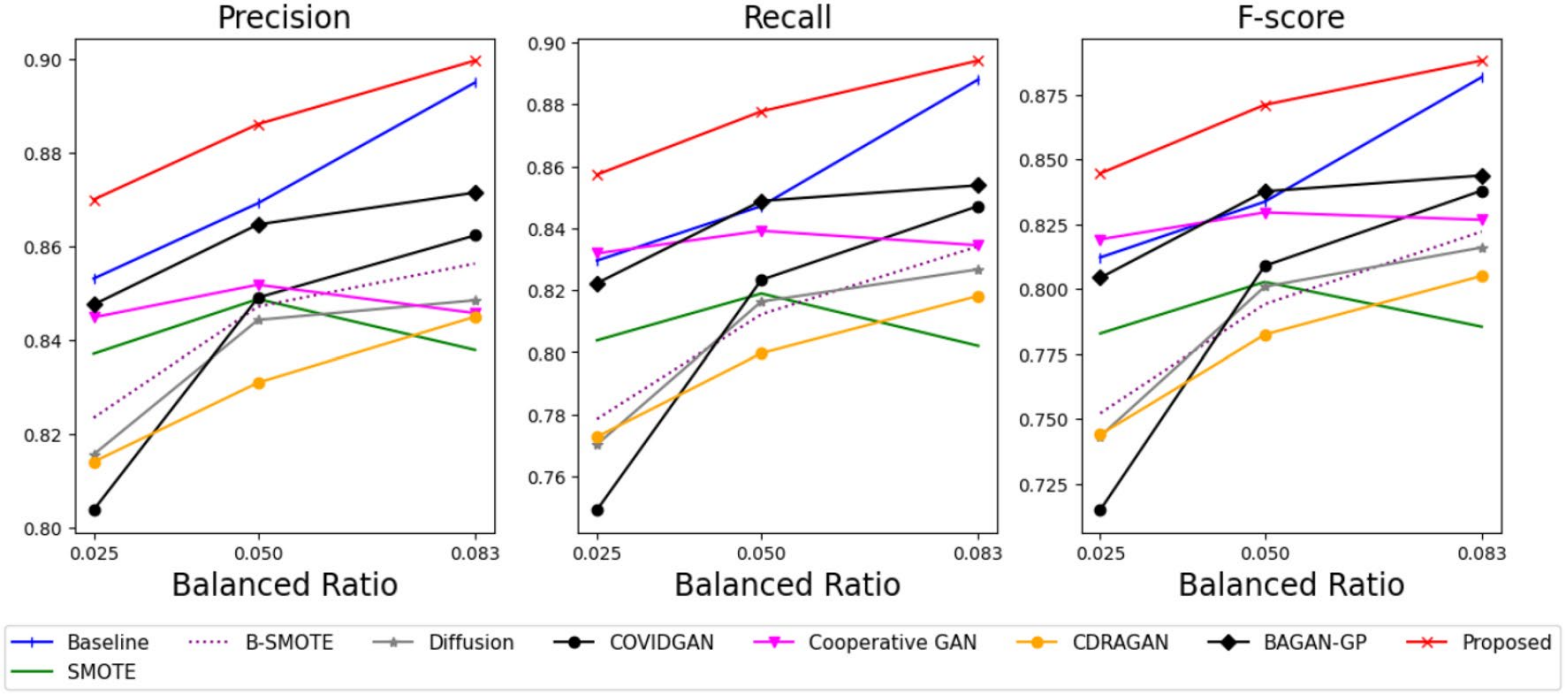
Performance evaluation using Alpha-Cyclodextrin hydrogel with several balanced ratios.

Figure 7 shows the performance evaluation of the benchmark and our methods using the generated samples from each method. The detailed averages and standard deviations of the performance of each method are provided in Appendix 1.4. In addition, Table 5 represents the average training time from each data augmentation method.

In this case studies, all benchmark methods represent worse results than the baseline. This might be caused by high similarities between the samples from the gel and solution phases, as shown in Fig. 5. It causes a challenging task. Therefore, the sampling-based methods that consider only local information offer inferior performance. Specifically, BAGAN-GP, CDRAGAN, Covid GAN, and class-conditioned diffusion model represent inferior results since the methods only focus on generating realistic samples but did not consider learning the phase-distinguishable features. Finally, Cooperative GAN also shows poor performance because of its unstable learning, resulting in a limited diversity of generated samples. Our method delivers the best performance by generating realistic and phase-distinguishable samples with a stabilizing technique. Specifically, our method improves 8.2%, 4.6%, and 7.0% of the average performance of the benchmark methods regarding their F-score, Precision, and Recall, respectively. However, the proposed method could not achieve statistically significant improvements over the best benchmark method, unlike the case studies using Pluronic F-127 hydrogel. This is because of the extremely high similarity between the solution and gel phases of Alpha-Cyclodextrin hydrogel, as shown in Fig. 5. However, the proposed method still achieves the best performance, while all the benchmark methods fail to generate suitable data. Therefore, it is still valuable to use the proposed method in such challenging data, although it still requires some computational resources, as shown in Table 5.

**Table 5:** On average training time of each method in Alpha-Cyclodextrin hydrogel case studies.

Method	Time (min)	Method	Time (min)	Method	Time (min)	Method	Time (min)
Proposed	22	Cooperative GAN	16	COVID GAN	3	SMOTE	0.5
BAGAN-GP	21	CDRAGAN	19	CCU-diffusion	16	BSMOTE	0.5

**Figure 8:**
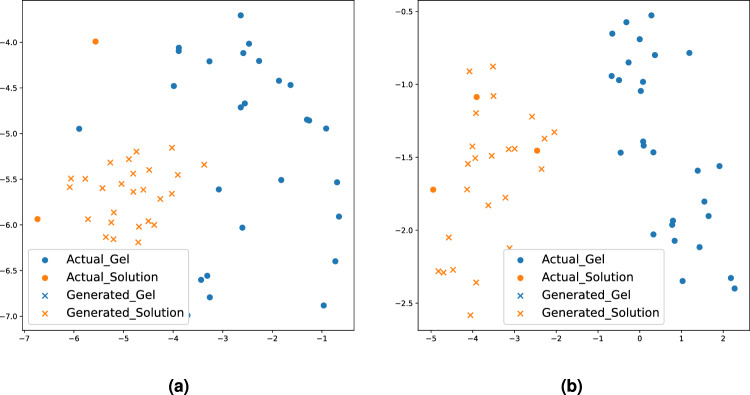
t-SNE of the feature from the intermediate layer of the classifier from our method in epochs (**a**) 0 and (**b**) 135 when the balanced ratio is 0.050.

Figure 8 illustrates the t-SNE visualization of the features extracted from the intermediate layer of classifiers in our method at epochs 0 and 135 when the balanced ratio is 0.050. Similar to Fig. 4, ‘$\bullet$’ and ‘$\times$’ denote features of actual and generated samples, respectively. To make a balanced training data, the solution phase of the Alpha-Cyclodextrin hydrogel has plenty of generated samples (‘$\times$’) than actual samples (‘$\bullet$’) in each batch. In contrast to epoch 0 (Fig. 8a), the features at epoch 135 (Fig. 8b) demonstrate that the features extracted from the generated samples (‘$\times$’) of the solution phase of the Alpha-Cyclodextrin hydrogel accurately match those from the actual samples (‘$\bullet$’). Due to this alignment, the balanced training data generated from our method achieves the best classification results compared to benchmark methods.

## Discussions

This paper addresses the material characteristics classification problem using imbalanced spectral data. The imbalanced spectral data usually happens in actual experiments and industries, causing poor supervised classification performance. To address this challenge, a GAN-based data augmentation method proposed by authors in the previous work^15^ is utilized. Specifically, the method consists of three DNNs, namely, generator, discriminator, and classifier, jointly optimized. The generator in the method generates both realistic and characteristics-distinguishable data to balance the training data. The imbalanced spectral data between the phases of Pluronic F-127 hydrogel and Alpha-Cyclodextrin hydrogel are used for the case studies. The results show the method successfully addresses the data imbalance problem by improving the phase classification results. Specifically, our method improves 8.8%, 6.4%, and 6.2% of the average performance of the benchmark methods regarding their F-score, Precision, and Recall, respectively, in all case studies. The outstanding performances of the proposed method in various case studies validate that the method could significantly contribute to many applications area using spectral data, such as radiology^45^ and additive manufacturing^46^. In addition, it would be an interesting future research topic to generate the minority data from the test data for the users who need to assess the performance of the methods requiring balanced test data.

## Methodology

A detailed procedure for the data collection of Pluronic F-127 and Alpha-Cyclodextrin hydrogel libraries are provided in “Data collection of Pluronic F-127 hydrogel libraries” and“Data collection of Alpha-Cyclodextrin hydrogel libraries” sections, respectively. Then, the proposed methodology is described in “Proposed methodology” section. Finally, the hyperparameters and the structure of the deep neural network used in this paper are listed in “Hyperparameters of the deep neural networks” section.

### Data collection of Pluronic F-127 hydrogel libraries

For the data collection, hydrogel libraries of Pluronic F-127 (PF-127) are obtained from Sigma Aldrich and are prepared in 96-well plates^47^. The stock PF-127 water solution (30% wt%) is first prepared with deionized water. The stock solution is then serial diluted with deionized water across the well plate for concentrations from 0.3125 wt% to 29.6875 wt%. The well plate is left in the fridge overnight for mixing. Then, the plate is taken out from the fridge and leave at room temperature in an hour for cross-linking. Next, the prepared PF-127 hydrogel libraries are characterized by piezoelectric milli-cantilever (PEMC) sensors. The PEMC sensor is integrated with a three-axis robot (MPS50SL; Aerotech), and its movement is controlled by a motion controller (A3200, Aerotech). The impedance spectrum of each hydrogel sample is captured by a network analyzer (E5061B, Keysight) and a customized MATLAB program. Spectra data of all PF127 hydrogels in the 96-well plates are collected by manually controlling the robot-integrated sensor to move from one well to another. The frequency range for each experiment and concentration is determined based on the spectrum width. In addition, different sensors are used in three repeated experiments, leading to varying spectrum frequency ranges. The frequency ranges span 26,013.75–37,000 Hz, 27,016.25–40,000 Hz, and 31,012.5–41,000 Hz for three repeated experiments, respectively. Finally, in the case of labeling, the spectral data are fitted to the sigmoid curve, and then the spectrum before the inflection point of the curve is labeled as a solution, and the spectrum after the inflection point is labeled as a gel.

### Data collection of Alpha-Cyclodextrin hydrogel libraries

To generate samples, supramolecular hydrogels of Alpha-Cyclodextrin ($\alpha$-CD)/Polyethylene glycol (PEG) are prepared in 96-well plates. Both $\alpha$-CD and PEG are obtained from Sigma Aldrich and used without further purification. Stock solutions of $\alpha$-CD (80 mg/mL) and PEG (240 mg/mL) are prepared in advance, and the hydrogel library is obtained by mixing a constant volume of PEG stock solution with different volumes of $\alpha$-CD stock solution and deionized water. At first, 190 $\upmu$L of PEG is pipetted into each well of the 96-well plate. Then, deionized water is pipetted by increasing from 95 to 190 $\upmu$L with a step size of 1 $\upmu$L. Next, $\alpha$-CD is pipetted by reducing from 190 to 95 $\upmu$L with a step size of 1 $\upmu$L. The final volume in each well is 380 $\upmu$L, and the concentration of PEG is 120 mg/mL, while the concentration of $\alpha$-CD varies from 20 to 40 mg/mL. To avoid the formation of inhomogeneous hydrogels, the precursor solution in each well is mixed by pipette immediately once $\alpha$-CD is added. After all wells have been formulated, the 96-well plate is further mixed by a digital shaker (LSE digital microplate shaker; Corning) at 1000 rpm for 10 min. Finally, the well plate was placed in a humid environment and reacted at room temperature for 12 h. Then, the prepared hydrogel libraries of $\alpha$-CD/PEG are characterized by PEMC sensors in a high-throughput manner. The PEMC sensor is integrated with a robot (FISNAR, F5200N) for automated characterization. The hydrogel in each well is characterized by penetrating the robot-integrated sensor into the sample, and the impedance spectra are collected by a network analyzer (E5061B, Keysight) and a customized MATLAB program. All samples in 96-well plates are automatically characterized by PEMC sensors with the computer-controlled robot. Finally, the phases of the collected $\alpha$-CD/PEG spectrum data are obtained by two best-fit linear regression models. Specifically, based on the point where the two linear regression models intersect, the spectrum before the point is identified as a solution and the spectrum after that as a gel.

### Proposed methodology

This section introduces a novel GAN-based data augmentation method proposed in the authors’ previous paper^15^. The structure of the overall method is described in “Three-player structure for imbalanced data learning” section. In addition, the objective functions of the algorithm are illustrated in “Objective functions for three-player” section. Finally, the training procedure of the method is described in “Training procedure” section.

#### Three-player structure for imbalanced data learning

**Figure 9:**
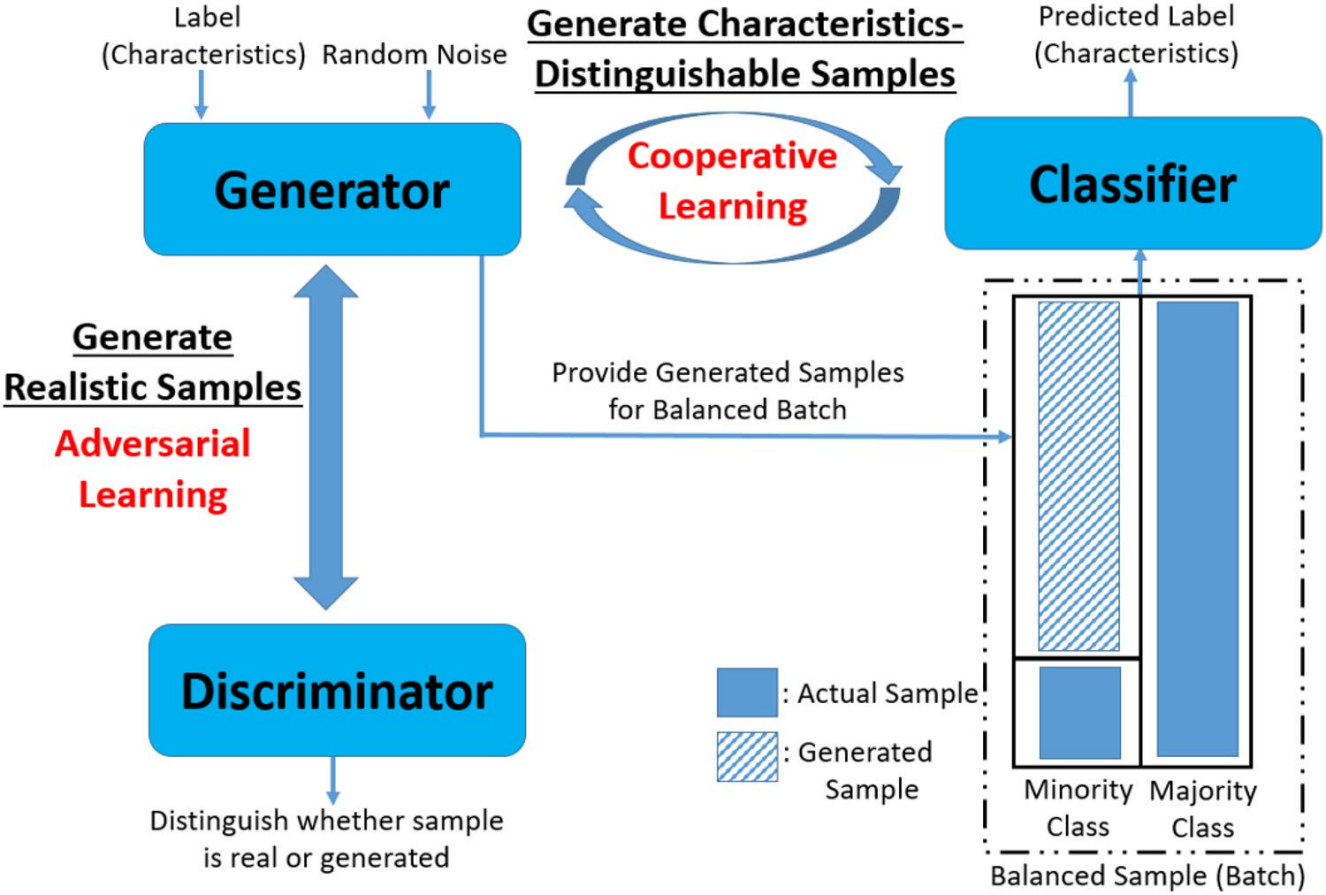
Structure of the method^15^.

Figure 9 shows the structure of our method, which consists of three players: a discriminator, a generator, and a classifier.

The generator generates samples of the spectral data using the random noise and corresponding characteristics labels. Within the generated samples, those representing the minority class are integrated with the actual imbalanced spectral data, resulting in balanced training data for the classifier. The proposed approach provides adversarial and cooperative learning to enhance the utility of the generated samples for improving the classifier’s performance. The specific roles of these two learnings are outlined as follows.

- Adversarial learning: The interaction between the generator and the discriminator adheres to the adversarial relationship inherent in the GAN structure. The relationship allows both networks to engage in a competitive process, ultimately leading to the generator’s generation of realistic spectral data.
- Cooperative learning: The cooperative interaction between the classifier and the generator empowers the generator to produce spectral data that can be well discerned regarding the material’s characteristics (i.e., characteristics-distinguishable samples) by the classifier.

Based on these two relationships, the generator generates samples of minority class with both properties (i.e., realistic and characteristics-distinguishable). Subsequently, these generated samples are combined with actual ones, creating a balanced training batch that flows through the classifier network in one training iteration. Through the iterative learning process involving three players, the classifier eventually attains a high level of performance. The detailed objective function of each player and the training procedure are explained in Appendix 1.

### Hyperparameters of the deep neural networks

Table 6 describes the hyperparameters that are used for all the methods in this paper. The common parameters among methods consisting of deep neural networks are determined based on the literature. Specifically, the optimizer of neural networks is the Adam algorithm with a learning rate of 0.0002 and momentums of 0.5 and 0.9^15^. In addition, many other hyperparameters, including kernel sizes, strides, padding, activation functions, and kernel initializer, are utilized by Huang and Jafari^28^ that proposed CDRAGAN and BAGAN-GP, which are the state-of-the-art class conditional GAN methods. Furthermore, the number of kernels is determined as two to the powers, including 32, 64, and 128, commonly used in the existing studies using convolutional neural networks^48,49^.

The unique parameters of each method are selected based on the guidelines provided in the literature or determined by the values that showed the best performance within a specific range. For example, the coefficient of the gradient penalty of BAGAN-GP and CDRAGAN are determined at ten based on the recommendation of the previous studies^28,50^ In the case of Cooperative GAN^36^, the scheduling parameter related to adjusting the borderline between classes is selected based on the performance from a range provided by Choi et al.^36^ ((0, 1]). For the SMOTE^16^ and B-SMOTE^17^, the parameters defining the number of neighborhood samples to use to generate the synthetic samples are selected based on the performance within a specified range ([1, 5]). Similarly, the dimension of the latent vector, which is the input size of the generator in Covid GAN^29^, is tuned within a range [100, 200]. From the class-conditioned U-Net based diffusion model^38,39^, the number of timesteps relevant to overfitting and underfitting to training data is determined as 1000 based on the previous literature^37,39^.

**Table 6:** Hyperparameters of each method.

Methods	Parameters	Value
SMOTE, B-SMOTE	Range of the nearest K samples	[1, 5]
CDRAGAN, BAGAN-GP cooperative GAN proposed	Number of epochs	150
	Optimizer	Adam
	Learning rate	0.0002
	Momentum	$\beta _{1}=0.5, \beta _{2}=0.9$
	Hidden layers (Discriminator)	4 blocks of [Conv2D, LeakyRelu]
	Hidden layers (Generator)	4 blocks of [Conv2D-Transpose, LeakyRelu, BatchNormalization]
	Number of Kernels in each block (Discriminator)	(64,128,128,256)
	Number of Kernels in each block (Generator)	(128,128,64,Number of channel)
	Kernel sizes	(4,4)
	Strides	(2,2)
	Padding	Same
	Activation functions	LeakyRelu, Tanh
	Kernel initializer	Random normal (sd=0.02)
	Slope of Leaky Relu	0.2
CDRAGAN, BAGAN-GP proposed	Gradient penalty coefficient	10
Cooperative GAN	Range of scheduling parameter	(0, 1]
Covid GAN	Range of latent vector dimensions	[100, 200]
CCU-diffusion	Timesteps	1000
BAGAN-GP, Proposed	Epochs in pre-training	100

Table 7 provides information on the hyperparameters used for the classifier in the case studies. In case studies, a CNN is utilized as the classifier. To ensure a fair and consistent comparison, all the methods adopt the identical classifier configuration outlined in Table 7.

**Table 7:** Hyperparameters of the classifier.

Parameters	Value
Number of epochs	150
Optimizer	Adam
Learning rate	0.0002
Momentum	$\beta _{1}=0.5, \beta _{2}=0.9$
Hidden layers	4 blocks of [Conv2D, LeakyRelu]
Number kernels in each block	(32,32,128,256)
Kernel sizes	(4,4)
Strides	(2,2)
Padding	Same
Activation functions	Leaky Relu, Softmax
Kernel initializer	Random normal (sd=0.02)
Slope of Leaky Relu	0.2

## Data Availability

The datasets used and/or analysed during the current study available from the corresponding author on reasonable request (zkong@vt.edu).

## Appendix 1

### Objective functions for three-player

The review of the generative adversarial network (GAN) is described in “Generative adversarial network (GAN)’’ section initially. Then, the objective functions of the discriminator, generator, and classifier are illustrated in “Objective function of discriminator’’, “Objective function of generator’’ and “Objective function of classifier’’ sections, respectively. The iterative optimization between the three players ultimately yields the high-performance classifier from the imbalanced spectral data.

#### Generative adversarial network (GAN)

The idea of a GAN is to train two networks, namely, generator *G* and discriminator *D*, with a minimax game for *V*(*D*, *G*) demonstrated in Eq. (2)^51^.

2 $$\begin{aligned} \min _{G}\max _{D}V(D,G)=\mathbb {E}_{(x_{a},y_{a})\sim P(X_{a},Y_{a})}[\text {log}(D(x_{a},y_{a}))] +\mathbb {E}_{(z,y_{g})\sim P(Z,Y_{g})}[\text {log}(1-D(G(z,y_{g}),y_{g})], \end{aligned}$$

where *z* denotes the random noise, and $x_{a}$ is actual samples from spectral data. $y_{a}$ and $y_{g}$ are the labels of actual and generated spectral data, respectively. Specifically, the generator is to produce samples of spectral data *G*(*z*) from *z*. In contrast, the discriminator is to distinguish whether the origin of input samples is from the actual ($x_{a}$) or the generator (*G*(*z*)). In other words, the role of the discriminator is to distinguish the origin of the input samples, whereas the generator’s task is to create synthetic samples with the intention of deceiving the discriminator. This adversarial learning leads to the distribution of newly generated samples approaching the inherent distribution of the actual samples, $P(X_{a})$.

#### Objective function of discriminator

In the proposed approach, the discriminator aims to maximize Eq. (2) through adversarial learning with the generator. Specifically, the discriminator learns to distinguish the input ($x_{a},y_{a}$) and ($G(z,y_{g}),y_{g}$) are actual and generated, respectively. Furthermore, the method introduces two supplementary terms to ensure a stable learning process. This is done because GAN training is usually unstable and challenging to converge, resulting in the generator’s gradient explosions in adversarial learning^52,53^. First, our method ensures the regularization of the discriminator’s gradient by imposing a gradient penalty. The penalty enforces 1-Lipschitz continuity upon the discriminator. Second, the proposed approach incorporates an extra input for the discriminator, comprising the actual sample with a wrong label. This added task prevents the discriminator from distinguishing the origin of the input well before the generator successfully approximates the actual sample distribution of the spectral data. Otherwise, it causes unstable learning of GAN through exploding or vanishing the gradient of the generator^53,54^. In summary, the objective function of the discriminator ($L^{D}$) is as follows^15^.

3 $$\begin{aligned} \begin{aligned} L^{D}(Z, X_{a}, Y_{a}, Y_{g}, Y_{m})=&\underbrace{-\mathbb {E}_{(x_{a},y_{a})\sim P(X_{a},Y_{a})}[\text {log}(D(x_{a},y_{a}))]}_{\text {loss from actual sample in discriminator}} \underbrace{-\mathbb {E}_{(z,y_{g})\sim P(Z,Y_{g})}[\text {log}(1-D(G(z,y_{g}),y_{g})]}_{\text {loss from generated sample in discriminator}} \\&\underbrace{-\mathbb {E}_{(x_{a},y_{m})\sim P(X_{a},Y_{m})}[\text {log}(1-D(x_{a},y_{m})]}_{\text {loss from mislabeled sample in discriminator}} + \lambda \underbrace{\mathbb {E}_{(\hat{x},y_{a})\sim P(\hat{X},Y_{a})}[(\Vert \nabla _{(\hat{x},y_{a})}D(\hat{x},y_{a}) \Vert _{2} -1)^{2}]}_{\text {loss from gradient penalty}}, \end{aligned} \end{aligned}$$

where $\hat{x}=\alpha x_{a} +(1-\alpha )G(z)$, and $\alpha$ is sampled uniformly between 0 and 1. The coefficient $\lambda$ pertains to the gradient penalty term. The initial three losses in Eq. (3) are associated with losses incurred when the discriminator misclassifies the source of the actual, generated, and mislabeled sample. The final loss corresponds to the loss linked to the gradient of the discriminator.

#### Objective function of generator

The primary aim of the generator is to generate samples that align with the distribution of actual spectral data ($P(X_{a})$), accomplished by minimizing Eq. (2). Hence, Eq. (2) enables the adversarial learning between the discriminator and generator. Apart from Eq. (2), the generator incorporates an additional component in its objective function that pertains to the classifier. Unlike the adversarial relationship with the discriminator, the generator and the classifier establish a cooperative relationship to generate distinctly discernible spectral samples across the material characteristics. In other words, the generator’s role is to generate samples and provide a balanced training dataset that can improve the classifier’s performance, as shown in Fig. 9. To accomplish this, the generator’s objective function includes the classification loss based on the generated samples. The objective function of the generator ($L^{G}$) can be formulated as follows^15^.

4 $$\begin{aligned} \begin{aligned} L^{G}(Z, Y_{g}) = \underbrace{-\mathbb {E}_{(z,y_{g})\sim P(Z,Y_{g})}[\text {log}(D(G(z,y_{g}),y_{g}))]}_{\text {loss from generated sample in discriminator}} \underbrace{-\mathbb {E}_{(z,y_{g})\sim P(Z,Y_{g})}[ y_{g}\text {log}(C(G(z,y_{g})))]}_{\text {loss from generated sample in classifier}}. \end{aligned} \end{aligned}$$

#### Objective function of classifier

The objective function of the classifier includes the classification loss derived from both the actual and generated samples of the spectral data. As illustrated in Fig. 9, the generator’s samples are combined with actual samples to provide a balanced training dataset for each batch of the classifier. The classifier is then optimized by minimizing the classification loss for both the actual and generated samples. Finally, the classifier’s objective function ($L^{C}$) is listed as follows^15^.

5 $$\begin{aligned} \begin{aligned} L^{C}(Z, X_{a},Y_{a}, Y_{g}) = \underbrace{-\mathbb {E}_{(x_{a},y_{a}) \sim P(X_{a},Y_{a})}[y_{a}\text {log}(C(x_{a}))]}_{\text {loss from actual sample in classifier}} \underbrace{-\mathbb {E}_{(z,y_{g})\sim P(Z, Y_{g})}[y_{g}\text {log}(C(G(z,y_{g}))]}_{\text {loss from generated sample in classifier}}. \end{aligned} \end{aligned}$$

In particular, $-\mathbb {E}_{(z,y_{g})\sim P(Z, Y_{g})}[y_{g}\text {log}(C(G(z,y_{g}))]$, a common term in both Eqs. (4) and (5) enables cooperative learning between the generator and classifier.

### Training procedure

The three players are optimized alternatively. Initially, the discriminator undergoes training using a batch that includes both actual and generated samples, aiming to minimize Eq. (3). Subsequently, a batch containing only generated samples is employed to update the generator, focusing on minimizing Eq. (4). Finally, the classifier’s training involves minimizing Eq. (5) with balanced training data from all the classes. This process begins with sampling a batch from the actual data. Then, the generator generates the remaining samples from the minority class to ensure a balanced training set. The alternating training process continues until it reaches the specified number of predefined epochs.

### Performance evaluation in Pluronic F-127 hydrogel case study

Tables 8, 9, and 10 represent the performance evaluation using the Pluronic F-127 hydrogel when the balanced ratios of training data are 0.039, 0.027, and 0.013, respectively.

**Table 8:** Performance evaluation in Pluronic F-127 hydrogel case study when the balanced ratio is 0.039.

	Pluronic F-127 hydrogel		
	Precision	Recall	F-score
Baseline	0.820 (0.05)	0.902 (0.04)	0.834 (0.06)
SMOTE	0.821 (0.03)	0.907 (0.02)	0.841 (0.03)
B-SMOTE	0.824 (0.03)	0.910 (0.02)	0.845 (0.03)
CDRAGAN	0.858 (0.04)	0.932 (0.02)	0.880 (0.04)
BAGAN-GP	0.855 (0.06)	0.927 (0.03)	0.875 (0.05)
Cooperative GAN	0.853 (0.05)	0.925 (0.03)	0.872 (0.05)
Covid GAN	0.831 (0.06)	0.912 (0.05)	0.846 (0.05)
CCU-diffusion	0.845 (0.05)	0.922 (0.03)	0.867 (0.05)
Proposed	0.923 (0.07)	0.946 (0.04)	0.926 (0.07)

Averages and standard deviations (in the parenthesis) are represented.

**Table 9:** Performance evaluation in Pluronic F-127 hydrogel case study when the balanced ratio is 0.027.

	Pluronic F-127 hydrogel		
	Precision	Recall	F-score
Baseline	0.795 (0.04)	0.886 (0.04)	0.805 (0.06)
SMOTE	0.792 (0.04)	0.880 (0.05)	0.799 (0.07)
B-SMOTE	0.793 (0.04)	0.881 (0.05)	0.800 (0.07)
CDRAGAN	0.818 (0.06)	0.900 (0.06)	0.828 (0.09)
BAGAN-GP	0.833 (0.07)	0.908 (0.06)	0.844 (0.09)
Cooperative GAN	0.800 (0.06)	0.883 (0.06)	0.806 (0.09)
Covid GAN	0.819 (0.05)	0.905 (0.05)	0.833 (0.07)
CCU-diffusion	0.849 (0.04)	0.831 (0.05)	0.821 (0.05)
Proposed	0.866 (0.06)	0.934 (0.03)	0.885 (0.06)

Averages and standard deviations (in the parenthesis) are represented.

**Table 10:** Performance evaluation in Pluronic F-127 hydrogel case study when the balanced ratio is 0.013.

	Pluronic F-127 hydrogel		
	Precision	Recall	F-score
Baseline	0.760 (0.05)	0.848 (0.07)	0.746 (0.10)
SMOTE	0.760 (0.05)	0.847 (0.07)	0.745 (0.11)
B-SMOTE	0.760 (0.05)	0.847 (0.07)	0.745 (0.11)
CDRAGAN	0.781 (0.07)	0.866 (0.06)	0.776 (0.09)
BAGAN-GP	0.784 (0.06)	0.867 (0.07)	0.778 (0.11)
Cooperative GAN	0.786 (0.06)	0.873 (0.06)	0.788 (0.09)
Covid GAN	0.761 (0.06)	0.846 (0.07)	0.743 (0.09)
CCU-diffusion	0.788 (0.08)	0.871 (0.06)	0.783 (0.10)
Proposed	0.836 (0.05)	0.919 (0.04)	0.855 (0.06)

Averages and standard deviations (in the parenthesis) are represented.

### Performance evaluation in Alpha-Cyclodextrin hydrogel case study

Tables 11, 12 and 13 represent the performance evaluation using the Alpha-Cyclodextrin hydrogel when the balanced ratios of training data are 0.083, 0.050, and 0.025, respectively.

**Table 11:** Performance evaluation in Alpha-Cyclodextrin hydrogel case study when the balanced ratio is 0.083.

	Alpha-Cyclodextrin hydrogel		
	Precision	Recall	F-score
Baseline	0.895 (0.02)	0.886 (0.02)	0.882 (0.05)
SMOTE	0.837 (0.03)	0.802 (0.05)	0.785 (0.06)
B-SMOTE	0.856 (0.03)	0.834 (0.06)	0.822 (0.06)
CDRAGAN	0.845 (0.04)	0.818 (0.05)	0.804 (0.06)
BAGAN-GP	0.871 (0.03)	0.858 (0.04)	0.843 (0.05)
Cooperative GAN	0.846 (0.03)	0.834 (0.03)	0.827 (0.03)
Covid GAN	0.761 (0.06)	0.847 (0.04)	0.837 (0.04)
CCU-diffusion	0.848 (0.03)	0.826 (0.03)	0.816 (0.05)
Proposed	0.900 (0.03)	0.894 (0.03)	0.888 (0.04)

Averages and standard deviations (in the parenthesis) are represented.

**Table 12:** Performance evaluation in Alpha-Cyclodextrin hydrogel case study when the balanced ratio is 0.050.

	Alpha-Cyclodextrin hydrogel		
	Precision	Recall	F-score
Baseline	0.869 (0.04)	0.847 (0.06)	0.834 (0.07)
SMOTE	0.848 (0.03)	0.819 (0.04)	0.794 (0.06)
B-SMOTE	0.847 (0.03)	0.812 (0.06)	0.803 (0.06)
CDRAGAN	0.830 (0.03)	0.800 (0.05)	0.782 (0.06)
BAGAN-GP	0.864 (0.03)	0.849 (0.04)	0.838 (0.05)
Cooperative GAN	0.851 (0.04)	0.839 (0.05)	0.830 (0.06)
Covid GAN	0.848 (0.02)	0.823 (0.04)	0.808 (0.05)
CCU-diffusion	0.844 (0.03)	0.816 (0.05)	0.800 (0.06)
Proposed	0.886 (0.03)	0.878 (0.04)	0.871 (0.04)

Averages and standard deviations (in the parenthesis) are represented.

**Table 13:** Performance evaluation in Alpha-Cyclodextrin hydrogel case study when the balanced ratio is 0.025.

	Alpha-Cyclodextrin hydrogel		
	Precision	Recall	F-score
Baseline	0.853 (0.04)	0.830 (0.05)	0.812 (0.06)
SMOTE	0.837 (0.02)	0.803 (0.04)	0.752 (0.05)
B-SMOTE	0.823 (0.03)	0.778 (0.05)	0.782 (0.06)
CDRAGAN	0.814 (0.05)	0.772 (0.08)	0.743 (0.10)
BAGAN-GP	0.847 (0.03)	0.822 (0.04)	0.804 (0.05)
Cooperative GAN	0.844 (0.05)	0.831 (0.06)	0.819 (0.07)
Covid GAN	0.803 (0.05)	0.749 (0.08)	0.714 (0.10)
CCU-diffusion	0.815 (0.03)	0.770 (0.06)	0.743 (0.07)
Proposed	0.870 (0.02)	0.857 (0.03)	0.846 (0.04)

Averages and standard deviations (in the parenthesis) are represented.

## References

[1] Friedrich W, Knipping P, Laue M (1913). Interferenzerscheinungen bei roentgenstrahlen. Ann. Phys..

[2] Callaghan PT (1993). Principles of Nuclear Magnetic Resonance Microscopy.

[3] Smith E, Dent G (2019). Modern Raman Spectroscopy: A Practical Approach.

[4] Wang H (2020). Rapid identification of X-ray diffraction patterns based on very limited data by interpretable convolutional neural networks. J. Chem. Inf. Model..

[5] Schuetzke J, Szymanski NJ, Reischl M (2023). Validating neural networks for spectroscopic classification on a universal synthetic dataset. NPJ Comput. Mater..

[6] Belsky A, Hellenbrandt M, Karen VL, Luksch P (2002). New developments in the inorganic crystal structure database (icsd): Accessibility in support of materials research and design. Acta Crystallogr. Sect. B Struct. Sci..

[7] Armbruster, T. & Danisi, R. The power of databases: The rruff project. *Highlights in Mineralogical Crystallography* 1–30 (2015).

[8] Schuetzke J, Benedix A, Mikut R, Reischl M (2021). Enhancing deep-learning training for phase identification in powder X-ray diffractograms. IUCrJ.

[9] Choudhary K (2022). Recent advances and applications of deep learning methods in materials science. NPJ Comput. Mater..

[10] Szymanski NJ (2021). Toward autonomous design and synthesis of novel inorganic materials. Mater. Horizons.

[11] McCulloch WS, Pitts W (1943). A logical calculus of the ideas immanent in nervous activity. Bull. Math. Biophys..

[12] Kantz ED, Tiwari S, Watrous JD, Cheng S, Jain M (2019). Deep neural networks for classification of lc-ms spectral peaks. Anal. Chem..

[13] Zeng, F., Peng, W., Kang, G., Feng, Z. & Yue, X. Spectral data classification by one-dimensional convolutional neural networks. In *2021 IEEE International Performance, Computing, and Communications Conference (IPCCC)* 1–6 (IEEE, 2021).

[14] Lee J-W, Park WB, Lee JH, Singh SP, Sohn K-S (2020). A deep-learning technique for phase identification in multiphase inorganic compounds using synthetic xrd powder patterns. Nat. Commun..

[15] Chung J, Shen B, Kong ZJ (2023). Anomaly detection in additive manufacturing processes using supervised classification with imbalanced sensor data based on generative adversarial network. J. Intell. Manuf..

[16] Chawla NV, Bowyer KW, Hall LO, Kegelmeyer WP (2002). Smote: Synthetic minority over-sampling technique. J. Artif. Intell. Res..

[17] Han, H., Wang, W.-Y. & Mao, B.-H. Borderline-smote: A new over-sampling method in imbalanced data sets learning. In *International Conference on Intelligent Computing* 878–887 (Springer, 2005).

[18] Cui W, Zhang Y, Zhang X, Li L, Liou F (2020). Metal additive manufacturing parts inspection using convolutional neural network. Appl. Sci..

[19] Lee XY, Saha SK, Sarkar S, Giera B (2020). Automated detection of part quality during two-photon lithography via deep learning. Addit. Manuf..

[20] Mycroft W (2020). A data-driven approach for predicting printability in metal additive manufacturing processes. J. Intell. Manuf..

[21] Douzas G, Bacao F (2018). Effective data generation for imbalanced learning using conditional generative adversarial networks. Expert Syst. Appl..

[22] Mikołajczyk, A. & Grochowski, M. Data augmentation for improving deep learning in image classification problem. In *2018 International Interdisciplinary PhD Workshop (IIPhDW)* 117–122 (IEEE, 2018).

[23] Fathy Y, Jaber M, Brintrup A (2020). Learning with imbalanced data in smart manufacturing: A comparative analysis. IEEE Access.

[24] Ranasinghe, G. D. & Parlikad, A. K. Generating real-valued failure data for prognostics under the conditions of limited data availability. In *2019 IEEE International Conference on Prognostics and Health Management (ICPHM)* 1–8 (IEEE, 2019).

[25] de Souza VLT, Marques BAD, Batagelo HC, Gois JP (2023). A review on generative adversarial networks for image generation. Comput. Graph..

[26] Sampath V, Maurtua I, Aguilar Martin JJ, Gutierrez A (2021). A survey on generative adversarial networks for imbalance problems in computer vision tasks. J. Big Data.

[27] Wang, C. *et al.* CGAN-plankton: Towards large-scale imbalanced class generation and fine-grained classification. In *2017 IEEE International Conference on Image Processing (ICIP)*, 855–859 (IEEE, 2017).

[28] Huang G, Jafari AH (2023). Enhanced balancing gan: Minority-class image generation. Neural Comput. Appl..

[29] Waheed A (2020). Covidgan: Data augmentation using auxiliary classifier gan for improved covid-19 detection. IEEE Access.

[30] Antoniou, A., Storkey, A. & Edwards, H. Data augmentation generative adversarial networks. Preprint at http://arxiv.org/abs/1711.04340 (2017).

[31] Kiyasseh D (2020). Plethaugment: Gan-based ppg augmentation for medical diagnosis in low-resource settings. IEEE J. Biomed. Health Inform..

[32] Mariani, G., Scheidegger, F., Istrate, R., Bekas, C. & Malossi, C. Bagan: Data augmentation with balancing gan. Preprint at http://arxiv.org/abs/1803.09655 (2018).

[33] Wu M (2021). Deep learning data augmentation for Raman spectroscopy cancer tissue classification. Sci. Rep..

[34] Gao B, Zhou J, Yang Y, Chi J, Yuan Q (2022). Generative adversarial network and convolutional neural network-based eeg imbalanced classification model for seizure detection. Biocybern. Biomed. Eng..

[35] Bisong, E. & Bisong, E. Google colaboratory. In *Building Machine Learning and Deep Learning Models on Google Cloud Platform: A Comprehensive Guide for Beginners* 59–64 (2019).

[36] Choi H-S, Jung D, Kim S, Yoon S (2021). Imbalanced data classification via cooperative interaction between classifier and generator. IEEE Trans. Neural Netw. Learn. Syst..

[37] Ho J, Jain A, Abbeel P (2020). Denoising diffusion probabilistic models. Adv. Neural Inf. Process. Syst..

[38] Sharma, G., Gupta, C., Agarwal, A., Sharma, L. & Dhall, A. Generating point cloud augmentations via class-conditioned diffusion model. In *Proc. IEEE/CVF Winter Conference on Applications of Computer Vision* 480–488 (2024).

[39] Nguyen, Q., Le, T., Nguyen, T. & Nhat, M. N. Class label conditioning diffusion model for robust brain tumor mri synthesis. *Authorea Preprints* (2023).

[40] Powers, D. M. Evaluation: From precision, recall and f-measure to roc, informedness, markedness and correlation. Preprint at http://arxiv.org/abs/2010.16061 (2020).

[41] Jalaal M, Cottrell G, Balmforth N, Stoeber B (2017). On the rheology of Pluronic f127 aqueous solutions. J. Rheol..

[42] Hsu, H. & Lachenbruch, P. A. *Paired t Test. Wiley StatsRef, Statistics Reference Online* (2014).

[43] Dimitriadis G, Neto JP, Kampff AR (2018). t-sne visualization of large-scale neural recordings. Neural Comput..

[44] Domiński A, Konieczny T, Kurcok P (2019). $$\alpha$$-cyclodextrin-based polypseudorotaxane hydrogels. Materials.

[45] Douek PC (2023). Clinical applications of photon-counting ct: A review of pioneer studies and a glimpse into the future. Radiology.

[46] Zhang W (2023). X-ray diffraction measurements and computational prediction of residual stress mitigation scanning strategies in powder bed fusion additive manufacturing. Addit. Manuf..

[47] Zhang J (2023). Rapid, autonomous high-throughput characterization of hydrogel rheological properties via automated sensing and physics-guided machine learning. Appl. Mater. Today.

[48] Naseri H, Mehrdad V (2023). Novel cnn with investigation on accuracy by modifying stride, padding, kernel size and filter numbers. Multimedia Tools Appl..

[49] Chang Y, Chen J, Qu C, Pan T (2020). Intelligent fault diagnosis of wind turbines via a deep learning network using parallel convolution layers with multi-scale kernels. Renew. Energy.

[50] Kodali, N., Abernethy, J., Hays, J. & Kira, Z. On convergence and stability of gans. Preprint at http://arxiv.org/abs/1705.07215 (2017).

[51] Wang, C., Yu, Z., Zheng, H., Wang, N. & Zheng, B. Cgan-plankton: Towards large-scale imbalanced class generation and fine-grained classification. In *2017 IEEE International Conference on Image Processing (ICIP)* 855–859 (IEEE, 2017).

[52] Tao, S. & Wang, J. Alleviation of gradient exploding in gans: Fake can be real. In *Proc. IEEE/CVF Conference on Computer Vision and Pattern Recognition* 1191–1200 (2020).

[53] Arjovsky, M. & Bottou, L. Towards principled methods for training generative adversarial networks. Preprint at http://arxiv.org/abs/1701.04862 (2017).

[54] Tran, N.-T., Bui, T.-A. & Cheung, N.-M. Dist-gan: An improved gan using distance constraints. In *Proc. European Conference on Computer Vision (ECCV)* 370–385 (2018).

